# Chitosan Modulates Volatile Organic Compound Emission from the Biocontrol Fungus *Pochonia chlamydosporia*

**DOI:** 10.3390/molecules28104053

**Published:** 2023-05-12

**Authors:** Jorge Mestre-Tomás, David Esgueva-Vilà, Alba Fuster-Alonso, Federico Lopez-Moya, Luis V. Lopez-Llorca

**Affiliations:** 1 Laboratory of Plant Pathology, Department of Marine Sciences and Applied Biology, University of Alicante, 03690 Alicante, Spainlv.lopez@ua.es (L.V.L.-L.); 2 Institute for Integrative Systems Biology (CSIC-UV), Spanish National Research Council, 46980 Paterna, Spain; 3 Institut de Ciències del Mar (ICM-CSIC), Renewable Marine Resources Department, 08003 Barcelona, Spain; afuster@icm.csic.es

**Keywords:** volatile organic compounds, chitosan, *Pochonia chalmydosporia*, gas chromatography, mass spectrometry

## Abstract

Fungal volatile organic compounds (VOCs) are responsible for fungal odor and play a key role in biological processes and ecological interactions. VOCs represent a promising area of research to find natural metabolites for human exploitation. *Pochonia chlamydosporia* is a chitosan-resistant nematophagous fungus used in agriculture to control plant pathogens and widely studied in combination with chitosan. The effect of chitosan on the production of VOCs from *P. chlamydosporia* was analyzed using gas chromatography–mass spectrometry (GC-MS). Several growth stages in rice culture medium and different times of exposure to chitosan in modified Czapek–Dox broth cultures were analyzed. GC-MS analysis resulted in the tentative identification of 25 VOCs in the rice experiment and 19 VOCs in the Czapek–Dox broth cultures. The presence of chitosan in at least one of the experimental conditions resulted in the *de novo* production of 3-methylbutanoic acid and methyl 2,4-dimethylhexanoate, and oct-1-en-3-ol and tetradec-1-ene in the rice and Czapek–Dox experiments, respectively. Other VOCs changed their abundance because of the effect of chitosan and fungal age. Our findings suggest that chitosan can be used as a modulator of the production of VOCs in *P. chlamydosporia* and that there is also an effect of fungal age and exposure time.

## 1. Introduction

There is an increasing incidence of pests in crops. This is aggravated by globalization and climate change [1]. The extensive use of phytosanitaries selects resistant pests, posing a threat to global food security [2]. Therefore, there is an increasing need for new tools that can be useful in different aspects of human life, such as volatile organic compounds (VOCs) [3,4,5].

The denomination of volatile organic compounds refers to organic compounds whose vapor pressure is at least 0.01 kPa at 20 °C [6]. Fungal VOCs are receiving increasing attention due to their potential in a wide variety of applications in the biotechnological sector, mainly in the field of agriculture, industry, and medicine [7,8]. Fungal VOCs can be used as biopesticides or as activators of plant defense [9,10]. They are also used in the perfume and food industries [8,11] and are a potential alternative to fossil fuels [12,13].

The origin of VOCs may determine the presence and amount of particular fungal volatiles available. This may, for instance, restrict their mass production. Therefore, finding new sources of VOC production could greatly facilitate their application in new fields. The production of fungal VOCs may be affected by growth medium, moisture, temperature, fungal growth, and growth modulators [8,14,15]. Here, we report the use of chitosan to modify VOC production by *Pochonia chlamydosporia* in composition and abundance.

*Pochonia chlamydosporia* (=*Metacordyceps chlamydosporia*) (Goddard) Zare and Gams 2001 (*Pc*) is a nematophagous fungus widely used as a biocontrol agent due to its ability to parasitize nematode eggs [16,17] and to induce systemic resistance and plant growth [18,19]. *Pc* is distributed worldwide and has a tritrophic (saprotrophic, endophytic, and nematophagous) lifestyle [20].

Chitosan is a linear biopolymer derived from the partial deacetylation of chitin consisting of β-(1→4)-*N*-acetyl-d-glucosamine and β-(1→4)-d-glucosamine [21]. Chitosan is a natural fungicide with antimicrobial activity [22] and an elicitor of plant defenses [23,24]. However, chitosan can promote the growth and sporulation of resistant fungi, such as *Pc*, or other fungal parasites of invertebrates, such as entomopathogenic fungi [25]. *Pc* can tolerate high chitosan doses and can degrade it and use it as a nutrient source [26]. *Pc* has enzymatic machinery, including a large number of chitosanases, which are induced during nematode egg parasitism [27,28]. Chitosan induces the chitosanolytic activity of *Pc* by modifying its gene expression [29].

Several studies have analyzed the ability of chitosan to induce VOCs in plants [30,31,32]. The production of these VOCs can be modulated with chitosan concentration and time of exposure [33,34]. The effect of chitosan on *Pc* at the molecular level has been widely studied [35]. However, to the best of our knowledge, no studies have been conducted on the effect of chitosan on VOC production by fungi.

The goal of this work was to determine the effect of chitosan on the production of VOCs by *Pochonia chlamydosporia* isolate 123. We investigated the influence of fungal growth and time of exposure to chitosan on VOC production. We focused on identifying which compounds were produced *de novo*, inhibited, or with a change in production with chitosan. Our hypothesis is that chitosan could be used as a modulator of the production of VOCs by *Pc*, leading to the discovery of new molecules that could have potential applications across multiple fields.

## 2. Results

We investigated whether the addition of chitosan to the culture medium, the age of the fungus culture, the time of exposure to chitosan, and the type of culture medium may influence the patterns of emission of volatile organic compounds by *P. chlamydosporia*.

### 2.1. Fungal VOC Profiles—Culture in Solid Medium (Rice)

**Figure 1:**
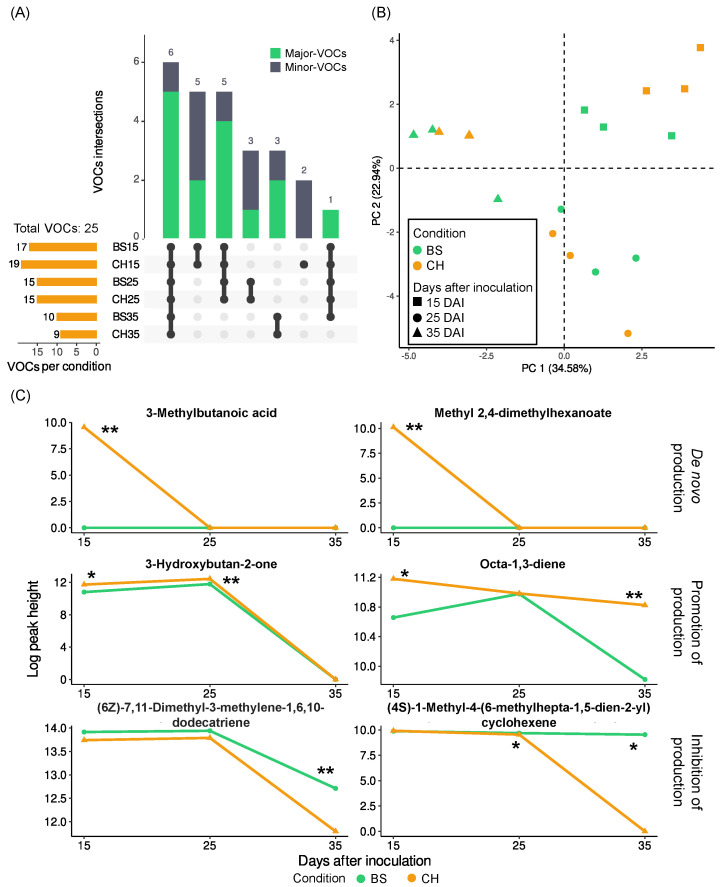
Effect of chitosan on VOC production by *P. chlamydosporia* on solid medium (rice grains). (**A**) UpSet plot of the intersection of VOCs found under culture conditions of *P. chlamydosporia* in rice. (**B**) PCA of the VOCs of *P. chlamydosporia* in rice. (**C**) Line plots with the median values of the log-transformed data (peak heights of VOCs + 1). Abbreviations: VOC = volatile organic compound; CH = chitosan treatment; BS = control buffer solution; * = Bayes Factor 3–10 or $\frac{1}{10}-\frac{1}{3}$; ** = Bayes Factor > 10 or <$\frac{1}{10}$.

*P. chlamydosporia* grown in Erlenmeyer flasks with rice yielded a total of 25 different VOCs in the three growth stages studied (15, 25, and 35 days after inoculation (DAI)) (Figure 1 and Figure S2). Based on tentative identification, the most abundant compounds detected for *Pc* grown on rice were 1,3-dimethoxybenzene, (6*Z*)-7,11-dimethyl-3-methylene-1,6,10-dodecatriene, and oct-1-en-3-ol (Table 1 and Table 2). Among the VOCs detected, 3-methylbutanoic acid and methyl 2,4-dimethylhexanoate were only found in the samples that were treated with chitosan, and 3-hydroxybutan-2-one, octa-1,3-diene, and five other VOCs were found to have increased in abundance at at least one of the different culture ages. In addition, (6*Z*)-7,11-Dimethyl-3-methylene-1,6,10-dodecatriene, (4*S*)-1-Methyl-4-(6-methylhepta-1,5-dien-2-yl)cyclohexene, and seven further VOCs showed a decrease at different fungal ages (Figure S1 and Table S1).

The PCA showed that samples were mainly grouped by fungal age, while for the 15-day old culture subgroup, there were also two associations, one for samples with chitosan and one for controls (Figure 1B). After 25 and 35 days of culture, controls and treatments were not well differentiated. Thus, the main differences in the grouping were mainly due to the culture age, although chitosan treatment seemed to also have an impact at each culture age.

When comparing the VOCs occurring in samples with chitosan and controls at different ages of the fungus, 19 VOCs were found in the youngest growth stage of *P. chlamydosporia* (15 days). Two minor VOCs were uniquely expressed when chitosan was present in the sample (3-methylbutanoic acid and methyl 2,4-dimethylhexanoate). In addition, four compounds showed moderate evidence of being emitted in higher abundance in the chitosan-treated samples than in the controls: 3-hydroxybutan-2-one (BF 10.08), octa-1,3-diene (BF 4.08), butane-2,3-diol (BF 3.79), and 1,2,3,4-tetramethoxybenzene (BF 6.32). On the other hand, 1,3-dimethoxybenzene (BF 0.31), 3,4-dimethoxyphenol (BF 0.13), and 1,3-dimethoxy-2-methylbenzene (BF 0.18) decreased their emissions, thus having higher abundance in the controls.

In the 25-day culture samples (second growth stage), 15 VOCs were found in total and shared in both conditions. However, four of them (3-hydroxybutan-2-one, methoxybenzene, 3-methoxyphenol, and 3,4-dimethoxyphenol) were more expressed in the samples with chitosan, and four other VOCs less so (octan-3-one, octan-3-ol, (1*S*,5*S*,6*R*)-2,6-Dimethyl-6-(4-methylpent-3-enyl)bicyclo[3.1.1]hept-2-ene, and (4*S*)-1-Methyl-4-(6-methylhepta-1,5-dien-2-yl)cyclohexene). 3-Hydroxybutan-2-one increased in abundance from 15 to 25 days of culture, but the difference between controls and chitosan-treated samples was greater, from 10.08 BF after 15 days to 41.33 BF after 25 days. On the other hand, 3,4-dimethoxyphenol went from having higher values in controls after 15 days of culture to having higher values in chitosan in a higher growth stage (25 days of culture).

Finally, in the oldest growth stage (35 days), the diversity of compounds was reduced to 10 VOCs, with 1 of which being only emitted in the absence of chitosan. The presence of chitosan resulted in an increase in only one compound, octa-1,3-diene, with a Bayes Factor of 14.22. On the other hand, five compounds showed a decrease in abundance in the presence of chitosan.

VOC diversity rapidly decreased as *P. chlamydosporia* aged, going from 19 VOCs after 15 days of growth to 10 VOCs after 35 days. Seven compounds were only emitted in the youngest stage of the fungus, two of which only when treated with chitosan. After 25 days, 15 different compounds were emitted, 2 of which were only emitted in this growth stage. Finally, after 35 days, six compounds from the previous stages and four new ones were released.

Of these compounds, octa-1,3-diene, methyl 2-phenylacetate, (4*S*)-1-Methyl-4-(6-methylhepta-1,5-dien-2-yl)cyclohexene, and 1,2,7,7-Tetramethylbicyclo[2.2.1]hept-2-ene showed a tendency to decrease with culture age. Furthermore, depending on whether the sample had been treated with chitosan or not, we found that the differences in abundance among the times markedly varied, as was the case of (4*S*)-1-Methyl-4-(6-methylhepta-1,5-dien-2-yl)cyclohexene, which had a less drastic decrease in controls than when treated with chitosan.

### 2.2. Fungal VOC Profiles—Culture in Liquid Medium (Modified Czapek–Dox)

**Figure 2:**
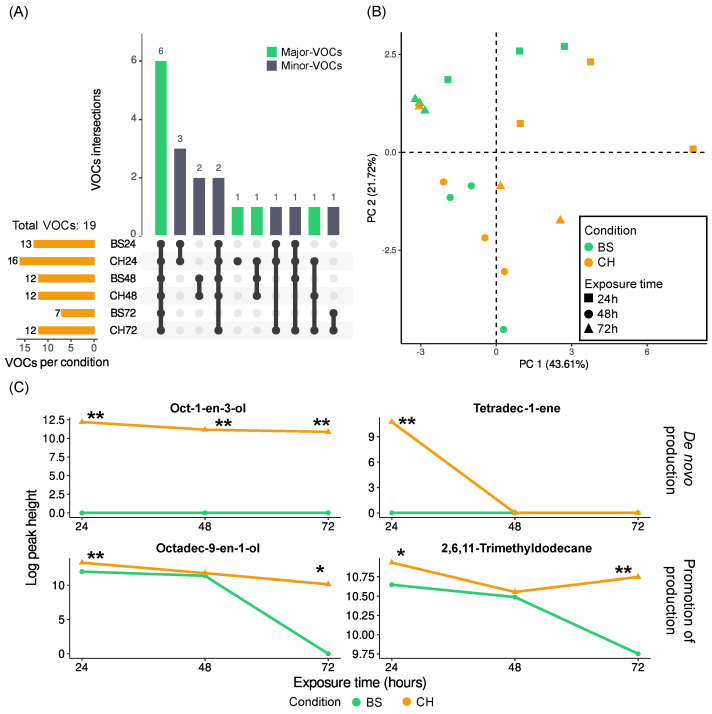
Effect of chitosan on VOC production by *P. chlamydosporia* in liquid medium (Czapek–Dox). (**A**) UpSet plot of the intersection of VOCs found under culture conditions of *P. chlamydosporia* in modified Czapek–Dox. (**B**). PCA of the VOCs from *P. chlamydosporia* in modified Czapek–Dox. (**C**) Line plots with the median values of the log-transformed data (peak heights of VOCs + 1). Abbreviations: VOC = volatile organic compound; CH = chitosan treatment; BS = control buffer solution; * = Bayes Factor 3–10 or $\frac{1}{10}-\frac{1}{3}$; ** = Bayes Factor > 10 or <$\frac{1}{10}$.

We also evaluated the effect of chitosan on VOC production by *Pc* in liquid medium (Czapek–Dox) (Figure S4). We detected a total of 19 VOCs, 2 of which (oct-1-en-3-ol and tetradec-1-ene) were only detected in the samples with chitosan (Figure 2). In addition, five VOCs were exclusively detected in the samples treated with chitosan at a specific exposure time (Table 3 and Table 4).

In the PCA, samples seemed to be more spread out than samples grown on rice (Figure 2B). However, we continued to see a tendency to cluster primarily by exposure time or age of culture, and within those big clusters, we found subgroups depending on whether the sample had been treated with chitosan or not.

When independently evaluating the three exposure times (24, 48, and 72 h in contact with chitosan), we saw that oct-1-en-3-ol was found all three times only when treated with chitosan. After five days of culture and 24 h with chitosan, we detected 16 different compounds (Figure S3 and Table S2), 3 of which were only present when treated with chitosan (oct-1-en-3-ol, 2,6,10,15-tetramethylheptadecane, and tetradec-1-ene) and 5 of which had higher abundance. Hexacosane (BF 21.89), hexadecan-1-ol (BF 150.67), and octadec-9-en-1-ol (BF 683.09) showed very high values of Bayes Factor. After two days with chitosan, controls and treatments showed fewer differences. We found 13 VOCs, of which oct-1-en-3-ol was only found in the chitosan samples, and 2,6,10-trimethyldodecane, in the controls; there were no differences in abundance for the rest of the VOCs. Finally, three days of the samples being in contact with chitosan was when the largest differences were found. Twelve VOCs were detected, five of which came only from chitosan-treated samples and four of which were produced in higher abundance. Five of the new or induced VOCs found after 72 h of exposure to chitosan were also detected at higher levels after 24 h of chitosan exposure (oct-1-en-3-ol, 8-methylheptadecane, 2,6,11-trimethyldodecane, hexacosane, and octadec-9-en-1-ol).

The comparison of VOCs among different exposure times revealed an increase in VOC diversity with longer exposure time or more culture days. The greatest differences were found between the treatment and the control after 3 days of exposure to chitosan. The abundance of compounds such as octadec-9-en-1-ol and hexadecan-1-ol tended to decrease when the time of exposure to chitosan was longer; however, the controls also decreased, though not in a such drastic way. There were other cases, such as 2,6,11-trimethyldodecane, nonadecane, 8-methylheptadecane, or 3,4,6-trimethylundecane, whose abundance decreased with time but remained almost constant when treated with chitosan.

## 3. Discussion

Volatile organic compounds (VOCs) are essential to many biological processes and have a wide range of uses from an anthropogenic perspective. Given the variety of industrial applications, many studies actually focus on VOCs from an economic perspective [8]. VOCs have good information-carrying properties, because they can disperse through the air and soil [36,37,38]. Fungi are capable of producing numerous VOCs, making their volatilomes a rich source of secondary metabolites [39]. In addition to the fungal species, other elements, such as the substrate, temperature, the age of the fungus, and the volatile extraction method itself, affect the composition of the volatilome [8]. Multiple studies have emphasized how chitosan acts as a defense inhibitor in plants and modifies secondary metabolism by triggering the synthesis of new VOCs [31,32,40]. The fungus *P. chlamydosporia* has exceptional enzymatic machinery for degrading chitosan and using it as a nutrient source [26,27,29]. To determine the changes in *Pc* volatilome when subjected to chitosan stress, the VOC profile was analyzed using HS-SPME/GC-MS in solid medium in different ages of the fungus and in liquid medium over different times of exposure to chitosan.

The findings indicate that chitosan could be a modulator of *Pc* secondary metabolism not only by triggering the production of new VOCs but also by increasing or decreasing the abundance of others. This experiment provides new insights into the chitosan modulatory effect that has already been observed in other organisms [32,33]. The compounds detected after applying chitosan may be due not only to the fungus-synthesized VOCs but also to substrate degradation products (either from rice, Czapek–Dox, or chitosan itself) [8]. *Pc* is a chitosan-resistant fungus [26] whose gene expression is modified when in contact with chitosan [29], inducing chitinases and chitosanases during nematode egg parasitism [27]. Together with other fungi that are resistant to chitosan, it is capable of producing valuable bioproducts by degrading chitosan [41]. This gene expression modification could be related to volatile production, suggesting the modulatory effect of chitosan on the volatilome of *Pc*.

In addition, the experiment with rice as a culture medium suggests that the volatilome evolves with the age of the fungus, which is in accordance with previous studies on other fungal species [10,15,42]. Pan et al. [42] analyzed the VOC profile of *Alternaria brassicae* and reported a reduction in the diversity of VOCs as the fungus aged, which is the same trend we found in *Pc*, where the 35-day-old culture had less VOC diversity than the 15-day-old culture. However, as Zhang et al. [15] noted, the early growth phases of *Neurospora dictyophora* (3–10 days of culture) reported fewer distinct VOCs than 15-day cultures, indicating that it may also be related to the fungal growth rate. The liquid-medium experiment suggests that the time of exposure to chitosan also has an effect on VOC production. After 72 h of exposure to chitosan, the volatilome was more similar to that after 48 h with or without chitosan than to that after 72 h without chitosan. Therefore, chitosan could slow down the age effect on the volatilome.

*Pc* produces compounds derived from benzene, hydrocarbons, ketones, and alcohol groups. VOCs found in *Pc* by Lozano-Soria et al. [10] were similar to but did not exactly match those determined in this study, possibly due to the different experimental conditions that affect VOC profile composition. In modified Czapek–Dox broth culture, oct-1-en-3-ol, which is a typical compound found in the odor of many fungi [43], was only detected in the presence of chitosan. Oct-1-en-3-ol has been reported to be a repellent of *Cosmopolites sordidus* [10] and has a role in fungal spore germination and dispersal [44]. Tetradec-1-ene was also only found in chitosan-treated cultures after 24 h of exposure, and a weak effect of attraction to the flour beetle *Tribolium confusum* has been reported [45]. 3-Methylbutanoic acid was registered with chitosan treatment after 10 days of growth in rice and has a strong association with the inhibition of germination and mycelium growth in several fungi [46,47].

In conclusion, our findings suggest that the effect of chitosan, and the relationship with culture age and chitosan exposure time alter the VOCs produced by *Pochonia chlamydosporia*. Chitosan modified the production of VOCs in *P. chlamydosporia* and triggered the synthesis of others. Future research should be performed to understand how other chitosan-resistant fungi respond to chitosan as well as to investigate and exploit the bioproducts produced.

## 4. Materials and Methods

### 4.1. Fungi and Source of Chitosan

*Pochonia chlamydosporia* var. *chlamydosporia* (=*Metacordyceps chlamydosporia* var. *chlamydosporium*) isolate 123 (*Pc*) (ATCC No. MYA-4875; CECT No. 20929) from the Phytopathology Laboratory (University of Alicante) fungal collection was used for experiments. *Pc* was isolated from nematode (*Heterodera avenae*) infected eggs.

Chitosan T8 with a molecular weight of 70 kDa and a deacetylation degree of 90.1% was provided by Marine BioProducts GmbH (Bremerhaven, Germany) and produced using crustacean exoskeletons. Chitosan solution was prepared as described by Palma-Guerrero et al. [25]. Briefly, chitosan was dissolved in 0.25 mol · L${}^{-1}$ HCl, and the pH was adjusted to 5.6 with NaOH under constant stirring. The resulting solution was dialyzed for 48 h for salt removal and autoclaved (120 °C for 20 min).

Chitosan solutions (CH) for the experiments (1 and 5 mg·mL${}^{-1}$) were prepared using autoclaved distilled water (120 °C, 20 min). A control buffer solution (BS) was also prepared in the same manner, without addition of chitosan.

### 4.2. Experimental Design

To obtain a wide view of the effect of chitosan on the synthesis of volatile organic compounds (VOCs) from *Pc* and to analyze the whole volatilome, two experiments were conducted.

In the first experiment (solid medium), *Pc* conidia (a suspension of 10${}^{6}$ conidia in 5 mL of autoclaved distilled water) were inoculated into 100 mL Erlenmeyer flasks, each containing 25 g of hydrated rice as solid culture medium, previously autoclaved (120 °C, 20 min). Flasks were covered with cotton and aluminum foil and incubated at 23–25 °C with natural lightning for (i) 10, (ii) 20, or (iii) 30 days. After these periods, 5 mL of chitosan (1 mg·mL${}^{-1}$ final concentration) was added to each flask and mixed well. Flasks were incubated for 5 more days. Controls were prepared by adding 5 mL of the buffer solution instead of chitosan. Moreover, flasks with rice only, rice plus chitosan solution, and rice plus buffer were also prepared. The experiment was replicated 3 times.

In the second experiment (liquid cultures), *Pc* was cultured in 20 mL screw-capped vials with 5 mL of modified Czapek–Dox broth medium prepared as described by Olivares-Bernabeu and López-Llorca [48]. *Pc* conidia were inoculated at a final concentration of 10${}^{6}$ conidia·mL${}^{-1}$. Vials were covered with cotton and aluminum foil and incubated at 25 °C under shaking at 120 rpm. After 5 days, modified Czapek–Dox liquid medium was removed to only leave *Pc* mycelia in the vials. A volume of 5 mL of chitosan (5 mg·mL${}^{-1}$) was then added to vials with *Pc* mycelia. Vials were further incubated sealed (to avoid VOC losses) for 24, 48, or 72 h at 25 °C. Afterwards, vials were taken for GC-MS analysis. Controls had 5 mL of buffer solution instead of chitosan. The experiment was replicated 3 times.

### 4.3. SPME and GC-MS Analysis

*Pc* VOCs were analyzed using headspace solid-phase microextraction (HS-SPME) and gas chromatography–mass spectrometry (GC MS). The GC-MS analysis was conducted using Agilent 7890B GC System equipped with a GERSTEL multipurpose autosampler (MPS).

In the first experiment, which involved solid medium, 5 g of homogenized *Pc* culture was added to 20 mL GERSTEL vials for VOC analysis. The vials were then sealed and left to equilibrate for 15 min. In the second experiment, *Pc* was already grown in the vials containing the liquid medium with different times of exposure to chitosan.

The VOCs were absorbed for 15 min using SPME divinylbenzene/carbon wide range/polydimethylsiloxane (DVB/C-WR/PDMS) fiber (10 mm in length, 80 μm in phase thickness, and 23 in needle gauge) at 60 °C. The desorption of the VOCs from the fiber took 240 s (4 min) in the GC injector, which was operated in splitless mode at a temperature of 250 °C. After the removal of the SPME fiber, chromatography continued for 53 min. Separation was performed with an Agilent J&W DB-624 capillary GC column (30 m × 0.25 mm × 1.4 μm). The column oven was initially set at 40 °C and ramped up at a rate of 5 °C·min${}^{-1}$ to reach the maximum temperature of 230 °C, which was held for 10 min. The carrier gas, helium, was maintained at a constant flow rate of 1 mL·min${}^{-1}$. Mass spectra were obtained using electron impact ionization with a mass range of 25 to 400 atomic mass units (a.m.u.), a scan speed of 1.562 u·s${}^{-1}$, and a frequency of 3.8 scans·s${}^{-1}$. The ionization source temperature was 230 °C and worked at 70 eV. The quadrupole temperature was maintained at 150 °C.

### 4.4. Tentative Identification of VOCs

The tentative identification of VOCs was performed using the NIST11.L Mass Spectral database. VOCs whose match with the mass spectra library was less than 50% were discarded, while the rest were evaluated using Agilent MassHunter Qualitative Analysis Navigator B.08.00 software to overlay chromatograms. Those compounds under each condition of the experiment that were only found in one of the three replicates were eliminated. VOCs detected two or more times under the same condition were selected.

Moreover, to identify VOCs exclusively produced by *Pc*, we removed artifacts such as silicones and column bleeding from our sample analyses. Additionally, volatiles from samples with rice only with either chitosan or buffer were discarded from the samples, assuming that they had not been produced by *Pc*.

Data were directly calculated from the peak heights of total ion current (TIC) profiles. The remaining VOCs were classified into two groups according to their abundance: major VOCs (peak height ≥ 50,000 ppm) and minor VOCs (peak height < 50,000 ppm).

### 4.5. Statistical Analysis

As part of the exploratory analysis of the data, Principal Component Analysis (PCA) was performed using the “prcomp” function from the R stats package (version 4.2.3). The data were standardized before PCA.

Regarding statistical modeling, we propose a Generalized Linear Model (GLM) [49] for each volatile and time of study (DAI for the rice experiment and exposure time for the Czapek experiment). The variable of interest or dependent variable is the concentration, and the explanatory or independent variable is the control/treatment factor.

$$\begin{array}{c} Y_{ij}∼Gamma\left(\mu_{ij},\phi \right),i=1,\ldots ,n;j=1,2;\\ log\left(\mu_{ij}\right)=\beta_{0}+\alpha_{ij}d, \end{array}$$

where *i* is the number of observations; *j* is the factor’s level; $Y_{ij}$ is the concentration and follows a Gamma distribution; $\mu_{ij}$ is the mean of the distribution, which is linked to the linear predictor by the log-link function; and the linear predictor consists of an intercept $\beta_{0}$ and a fixed factor $\alpha_{ij}d$ (control/treatment).

Parameter and hyperparameter estimation was performed in the context of Bayesian inference [50,51]. For that, computational approaches and numerical approximations are needed to estimate the posterior distribution of parameters. In our study, we applied Integrated Nested Laplace Approximation (INLA) using R-INLA (version 23.03.19) to approximate the posterior marginal distributions of the model’s parameters and hyperparameters [52]. Finally, vaguely informative prior distributions were used for all the parameters.

Once we sampled the posterior distributions of $\mu_{ij}$, we computed the differences among them in order to analyze the effect of chitosan. Then, Bayesian hypothesis contrast was performed on the difference in these posterior distributions (θ). For that, we calculated the Bayes Factor (BF) using the coefficients of the probability of being different from 0 ($\alpha_{0}=P\left(\theta <0|data\right)$ and $\alpha_{1}=P\left(\theta >0|data\right)$). The BF quantifies the evidence of $H_{1}$ with respect to $H_{0}$, where large values of BF indicate greater support for $H_{1}$, and *vice versa* [53,54]. Jeffreys [53] established one of the best-known criteria for the interpretation of the Bayes Factor [55]. A Bayes Factor greater than 1 supports the alternative hypothesis, while a value less than 1 favors the null hypothesis. Bayes Factors between 1/3 and 3 are generally considered to provide barely worth mentioning evidence. Values between 3 and 10 (or between 1/10 and 1/3) indicate substantial evidence, those between 10 and 30 (or between 1/30 and 1/10) indicate strong evidence, and those above 30 (or below 1/30) indicate very strong evidence for one hypothesis over the other.

## 5. Conclusions

Our findings suggest that chitosan can modulate the production of volatile organic compounds (VOCs) by *P. chlamydosporia* and perhaps similar biocontrol fungi. This could be a novel approach to generate VOCs with various applications in agrobiotechnology and medical fields.

## 6. Patents

The results of this paper have been filed for a Spanish Patent (P202230103), with Jorge Mestre-Tomás, David Esgueva-Vilà, Federico Lopez-Moya, and Luis V. Lopez-Llorca as inventors.

## Figures and Tables

**Table 1:** Mean peak heights and standard deviations of VOCs detected in rice control samples of *P. chlamydosporia* after different culture times (mean (SD)). Abbreviations: VOC = volatile organic compound; DAI = days after inoculation.

	Control Buffer Solution		
**Major VOCs**	**15 DAI**	**25 DAI**	**35 DAI**
3-Hydroxybutan-2-one	66,402.67 (52,092.53)	125,554.33 (27,721.47)	0 (0)
Octa-1,3-diene	56,919.33 (41,951.78)	55,423.67 (53,923.95)	16,699 (5750.02)
Butane-2,3-diol	6820.33 (11,813.16)	0 (0)	0 (0)
Methoxybenzene	69,604 (29,288.09)	91,969.33 (50,952.5)	39,812.67 (50,272.28)
Oct-1-en-3-ol	649,414 (228,962.08)	0 (0)	0 (0)
Octan-3-one	0 (0)	399,307.33 (46,213.61)	0 (0)
Octan-3-ol	119,415 (42,769.72)	114,748 (41,654.88)	0 (0)
1,3-Dimethoxybenzene	30,228,196.67 (4,503,694.87)	23,852,155.67 (3,776,948.08)	24,519,265 (853,358.59)
Methyl 2-phenylacetate	21,108.33 (36,560.71)	26,653 (8728.81)	0 (0)
3-Methoxyphenol	57,144.67 (13,644.68)	82,675.67 (46,591.62)	176,919.67 (282,216.43)
1,2,3-Trimethoxybenzene	0 (0)	0 (0)	79,005 (70,432.6)
3,4-Dimethoxyphenol	20,412 (6363.42)	16,339.67 (4189.58)	47,431 (28,622)
1,2,4-Trimethoxybenzene	0 (0)	0 (0)	69,126.33 (59,929.47)
(1*S*,5*S*,6*R*)-2,6-Dimethyl-6-(4-methylpent-3-enyl)bicyclo[3.1.1]hept-2-ene	91,357 (9722.48)	95,971 (51,607.35)	0 (0)
(6*Z*)-7,11-Dimethyl-3-methylene-1,6,10-dodecatriene	1,102,849.33 (104,001.08)	1,402,859 (620,066.31)	442,461.67 (217,469.06)
	**Control Buffer Solution**		
**Minor VOCs**	**15 DAI**	**25 DAI**	**35 DAI**
3-Methylbutanoic acid	0 (0)	0 (0)	0 (0)
Hept-2-enal	0 (0)	7376.33 (12,776.18)	0 (0)
1,2,7,7-Tetramethylbicyclo[2.2.1]hept-2-ene	16,335.33 (28,293.63)	20,317 (8170.16)	0 (0)
Methyl 2,4-dimethylhexanoate	0 (0)	0 (0)	0 (0)
1,3-Dimethoxy-2-methylbenzene	14,198.33 (7099.4)	0 (0)	0 (0)
(4*S*)-1-Methyl-4-(6-methylhepta-1,5-dien-2-yl)cyclohexene	20,789 (2941.92)	21,290 (11,113.46)	14,563 (2446.21)
1,4-Dichloro-2,5-dimethoxybenzene	0 (0)	0 (0)	22,518.67 (22,153.03)
2,4-Dimethylquinoline	10,420.33 (3401.84)	0 (0)	0 (0)
1,2,3,4-Tetramethoxybenzene	20,149.33 (6534.38)	0 (0)	0 (0)
2,7,7,10-Tetramethyl-3-oxatetracyclo[7.3.0.0${}^{2,4}$.0${}^{6,8}$]dodecane	0 (0)	21,819.67 (8513.99)	0 (0)

**Table 2:** Mean peak heights and standard deviations of VOCs detected in chitosan-treated rice samples of *P. chlamydosporia* after different culture times (mean (SD)). Abbreviations: VOC = volatile organic compound; DAI = days after inoculation.

	Chitosan Solution		
**Major VOCs**	**15 DAI**	**25 DAI**	**35 DAI**
3-Hydroxybutan-2-one	140,203.67 (53,930.99)	388,898.33 (323,491.84)	0 (0)
Octa-1,3-diene	89,883.33 (40,449.43)	69,747 (19,223.05)	40,255.33 (22,192.96)
Butane-2,3-diol	38,923.67 (47,948.61)	0 (0)	0 (0)
Methoxybenzene	51,741.33 (13,805.67)	152,280.33 (17,822.66)	3329 (5766)
Oct-1-en-3-ol	399,266 (405,852.5)	0 (0)	0 (0)
Octan-3-one	0 (0)	277,246.67 (54,831.37)	0 (0)
Octan-3-ol	130,339.33 (39,439.22)	46,439.33 (15,604.4)	0 (0)
1,3-Dimethoxybenzene	23,396,956.33 (2,794,659)	27,827,414 (1,515,361.21)	8,751,248.67 (11,680,587.39)
Methyl 2-phenylacetate	48,876.67 (3305.18)	31,430.67 (5845.67)	0 (0)
3-Methoxyphenol	73,917 (28,675.39)	191,472.33 (20,750.74)	0 (0)
1,2,3-Trimethoxybenzene	0 (0)	0 (0)	31,746.67 (54,986.84)
3,4-Dimethoxyphenol	12,502 (3616.14)	25,793 (1228)	5007.67 (8673.53)
1,2,4-Trimethoxybenzene	0 (0)	0 (0)	25,044 (43,377.48)
(1*S*,5*S*,6*R*)-2,6-Dimethyl-6-(4-methylpent-3-enyl)bicyclo[3.1.1]hept-2-ene	84,528.33 (16,562.62)	44,375 (13,065.53)	0 (0)
(6*Z*)-7,11-Dimethyl-3-methylene-1,6,10-dodecatriene	922,376.67 (40,678.69)	1,068,108.67 (492,573.01)	176,422.67 (118,053.64)
	**Chitosan Solution**		
**Minor VOCs**	**15 DAI**	**25 DAI**	**35 DAI**
3-Methylbutanoic acid	14,420 (2770.17)	0 (0)	0 (0)
Hept-2-enal	0 (0)	16,133 (5651.12)	0 (0)
1,2,7,7-Tetramethylbicyclo[2.2.1]hept-2-ene	28,434.67 (10,528.13)	19,826.33 (3413.38)	0 (0)
Methyl 2,4-dimethylhexanoate	23,886.67 (1965.31)	0 (0)	0 (0)
1,3-Dimethoxy-2-methylbenzene	8232.33 (4957.03)	0 (0)	0 (0)
(4*S*)-1-Methyl-4-(6-methylhepta-1,5-dien-2-yl)cyclohexene	20,508 (3187.7)	14,716.33 (6302.52)	3672.67 (6361.25)
1,4-Dichloro-2,5-dimethoxybenzene	0 (0)	0 (0)	7982.67 (13,826.38)
2,4-Dimethylquinoline	11,458 (4963.33)	0 (0)	0 (0)
1,2,3,4-Tetramethoxybenzene	32,050 (11,831.09)	0 (0)	0 (0)
2,7,7,10-Tetramethyl-3-oxatetracyclo[7.3.0.0^2,4^.0^6,8^]dodecane	0 (0)	18,851 (22,189.55)	0 (0)

**Table 3:** Mean peak heights and standard deviations of VOCs detected in control *P. chlamydosporia* samples grown in modified Czapek–Dox for different exposure times (mean (SD)). Abbreviations: VOC = volatile organic compound.

	Control Buffer Solution		
**Major VOCs**	**24 h**	**48 h**	**72 h**
Oct-1-en-3-ol	0 (0)	0 (0)	0 (0)
8-Methylheptadecane	64,212.67 (27,072.87)	66,092.33 (31,020.32)	33,097 (8613.77)
2,6,11-Trimethyldodecane	38,103.67 (12,330.32)	34,817.33 (5539.97)	18,099.33 (4608.43)
Hexacosane	23,811.67 (7685.3)	64,745.33 (14,575.82)	24,891.33 (3341.71)
2,4-ditert-butylphenol	116,881 (39,599.25)	78,442.67 (730.06)	59,575.33 (3437.02)
2,6,10,15-Tetramethylheptadecane	0 (0)	42372.67 (16256.22)	0 (0)
Tetradec-1-ene	0 (0)	0 (0)	0 (0)
Hexadecan-1-ol	361,050.67 (110,953.32)	224,019.67 (135,154.82)	6539.67 (11,327.03)
Octadec-9-en-1-ol	171,950 (42,297.08)	90,861.33 (40,895.74)	4027.67 (6976.12)
	**Control Buffer Solution**		
**Minor VOCs**	**24 h**	**48 h**	**72 h**
2-Methylpyrazine	0 (0)	0 (0)	5531 (9579.97)
3,7-Dimethyldecane	15,702.67 (13,631.84)	0 (0)	0 (0)
5-Methylundecane	13,636 (11,954.55)	0 (0)	0 (0)
Naphthalene	0 (0)	10,350 (17,926.73)	0 (0)
1,3-ditert-butylbenzene	16,117.67 (14,093.38)	0 (0)	0 (0)
3,4,6-Trimethylundecane	12,117.67 (10,601.65)	5167 (8949.51)	0 (0)
2,6,10-Trimethyldodecane	4661 (8073.09)	9740 (8511.64)	0 (0)
3-Ethyl-5-(2-ethylbutyl)octadecane	9472.67 (8294.68)	0 (0)	0 (0)
Nonadecane	9776.67 (8620.81)	30,276.33 (6070.58)	0 (0)
2,6,11,15-Tetramethylhexadecane	0 (0)	13,774 (12,290.88)	0 (0)

**Table 4:** Mean peak heights and standard deviations of VOCs detected in *P. chlamydosporia* samples grown in modified Czapek–Dox for different times of exposure to chitosan (hours) (mean (SD)). Abbreviations: VOC = volatile organic compound.

	Chitosan Solution		
**Major VOCs**	**24 h**	**48 h**	**72 h**
Oct-1-en-3-ol	218,516.67 (130,830.14)	132,448.33 (113,897.66)	55,408.67 (24,658.18)
8-Methylheptadecane	87,610.33 (33,754.89)	69,603.33 (37,892.55)	74,636.33 (48,102.79)
2,6,11-Trimethyldodecane	59,529 (24,339.84)	35,308.67 (9317.39)	47,015.67 (30,486.18)
Hexacosane	51,634.33 (21,668.02)	58,443.33 (20,214.85)	59,734.67 (28,128.13)
2,4-ditert-butylphenol	131,087.33 (33,937.52)	69,674.33 (8951)	75,155.67 (33,691.57)
2,6,10,15-Tetramethylheptadecane	27,279.33 (26,850.31)	40,188.67 (5461)	0 (0)
Tetradec-1-ene	34,551.33 (30,503.11)	0 (0)	0 (0)
Hexadecan-1-ol	1,036,705 (126,868.82)	191,852.33 (29,836.23)	17,328.67 (15,497.53)
Octadec-9-en-1-ol	626,179.33 (96,866.49)	118,469 (33,443.61)	21,290.33 (6700.12)
**Minor VOCs**	**24 h**	**48 h**	**72 h**
2-Methylpyrazine	0 (0)	0 (0)	5023 (8700.09)
3,7-Dimethyldecane	11179.67 (9686.23)	0 (0)	0 (0)
5-Methylundecane	10,221 (8929.62)	0 (0)	0 (0)
Naphthalene	0 (0)	6897.67 (5980.28)	0 (0)
1,3-ditert-butylbenzene	25,663 (9942.73)	0 (0)	0 (0)
3,4,6-Trimethylundecane	16,235.67 (14,948.86)	10,693.33 (9270.68)	13,517.67 (12,817.11)
2,6,10-Trimethyldodecane	11,949 (11,228.94)	0 (0)	17,214.67 (16,102.73)
3-Ethyl-5-(2-ethylbutyl)octadecane	14,332.67 (13,385.78)	0 (0)	11,371.33 (10,643.96)
Nonadecane	27,725.33 (11,623.31)	27,063 (4893.47)	24,760 (21,735.48)
2,6,11,15-Tetramethylhexadecane	0 (0)	16,144 (949.76)	0 (0)

## Data Availability

The data presented in this study are available in Supplementary Materials.

## Supplementary Materials

The following supporting information can be downloaded at: https://www.mdpi.com/article/10.3390/molecules28104053/s1, Table S1: Bayes Factor between treatment and control of VOCs from *P. chlamydosporia* in rice cultures on different DAI, Table S2: Bayes Factor between treatment and control of VOCs from *P. chlamydosporia* in Czapek–Dox broth cultures after different times of exposure to chitosan, Figure S1: Line plots with the median values and standard deviations of the peak heights of VOCs produced by *P. chlamydosporia* in rice cultures, Figure S2: GC-MS chromatogram of headspace volatile compounds produced by *P. chlamydosporia* 15 days after inoculation with control buffer solution (BS) and with chitosan solution (CH), Figure S3: Line plots with the median values and standard deviations of the peak heights of VOCs produced by *P. chlamydosporia* in modified Czapek–Dox broth cultures, Figure S4: GC-MS chromatogram of headspace volatile compounds produced by *P. chlamydosporia* cultured in modified Czapek–Dox broth for 5 days after 24 h with control buffer solution (BS) and 24 h of exposure to chitosan (CH).

## Abbreviations

The following abbreviations are used in this manuscript:

*Pc*	*Pochonia chlamydosporia*
DAI	Days after inoculation
GC	Gas chromatography
HS-SPME	Headspace solid-phase microextraction
MS	Mass spectrometry
VOC	Volatile organic compound
