# Supplementary material for: Chitosan Modulates Volatile Organic Compound Emission from the Biocontrol Fungus Pochonia chlamydosporia
Source: Molecules. 2023 May 12;28(10):4053. doi: 10.3390/molecules28104053 (PMC10223989; doi:10.3390/molecules28104053)
Supplement: Supplementary file 1 [file molecules-28-04053-s001.zip › molecules-2358563-supplementary.pdf]

Supplementary material: Chitosan modulates volatile organic compound emission from the biocontrol fungus *Pochonia chlamydosporia*

Jorge Mestre-Tomás, David Esgueva-Vilà, Alba Fuster-Alonso,

Federico Lopez-Moya and Luis V. Lopez-Llorca

- **Supplemental data and code**: https://github.com/jmestret/PcVOCs (curated GC-MS datasets and R code for statistical analysis).

Table S1: Bayes Factor between treatment and control of VOCs from *P. chlamydosporia* in rice cultures at different DAI. "CH" means that was only present in samples treated with chitosan, "BS" that only appeared in controls and blank gaps that was not detected in any condition. Abbreviations: CH = Chitosan treatment, BS = Control buffer solution, 15 = 15 days after inoculation, 25 = 25 days after inoculation, 35 = 35 days after inoculation.

**Compound**	**CH15 - BS15**	**CH25 - BS25**	**CH35 - BS35**
3-Hydroxybutan-2-one	**10.08**	**41.33**	
Octa-1,3-diene	**4.08**	1.24	**19.94**
Butane-2,3-diol	**3.79**		
3-Methylbutanoic acid	**CH**		
Methoxybenzene	0.33	**6.38**	**0.15**
Hept-2-enal		1.81	
Oct-1-en-3-ol	0.66		
Octan-3-one		**0.19**	
1,2,7,7-Tetramethylbicyclo[2.2.1]hept-2-ene	1.54	0.91	
Octan-3-ol	1.4	**0.03**	
Methyl 2,4-dimethylhexanoate	**CH**		
1,3-Dimethoxybenzene	**0.31**	2.01	**0.1**
Methyl 2-phenylacetate	1.93	1.96	
3-Methoxyphenol	2.72	**22.49**	**T**
1,2,3-Trimethoxybenzene			0.76
3,4-Dimethoxyphenol	**0.13**	**7.77**	**0.18**
1,2,4-Trimethoxybenzene			0.86
(1*S*,5*S*,6*R*)-2,6-Dimethyl-6-(4-methylpent-3-enyl)bicyclo[3.1.1]hept-2-ene	0.71	**0.05**	
1,3-Dimethoxy-2-methylbenzene	**0.18**		
(6*Z*)-7,11-Dimethyl-3-methylene-1,6,10-dodecatriene	0.44	0.38	**0.05**
(4*S*)-1-Methyl-4-(6-methylhepta-1,5-dien-2-yl)cyclohexene	0.94	**0.28**	**0.3**
1,4-Dichloro-2,5-dimethoxybenzene			0.64
2,4-Dimethylquinoline	1.43		
1,2,3,4-Tetramethoxybenzene	**6.32**		
2,7,7,10-Tetramethyl-3-oxatetracyclo[7.3.0.0^{2,4}.0^{6,8}]dodecane		0.87	

Table S2: Bayes Factor between treatment and control of VOCs from *P. chlamydosporia* in Czapek-Dox broth cultures at different exposure times to chitosan. "CH" means that was only present in samples treated with chitosan, "BS" that only appeared in controls and blank gaps that was not detected in any condition. Abbreviations: CH = Chitosan treatment, BS = Control buffer solution, 24 = 24 hours of exposure time to chitosan, 48 = 48h exposure time, 72 = 72h exposure time.

**Compound**	**CH24 - BS24**	**CH48 - BS48**	**CH72 - BS72**
2-Methylpyrazine			0.67
Oct-1-en-3-ol	**CH**	**CH**	**CH**
3,7-Dimethyldecane	0.77		
5-Methylundecane	0.8		
Naphthalene		0.78	
1,3-ditert-butylbenzene	1.6		
8-Methylheptadecane	**3.06**	1.17	**15.97**
3,4,6-Trimethylundecane	1.25	1.58	**CH**
2,6,10-Trimethyldodecane	1.75	**T**	**CH**
2,6,11-Trimethyldodecane	**5.49**	1.06	**25.99**
3-Ethyl-5-(2-ethylbutyl)octadecane	1.37		**CH**
Hexacosane	**21.89**	0.66	**32.33**
Nonadecane	2.97	0.61	**CH**
2,4-ditert-butylphenol	1.57	0.58	2.52
2,6,10,15-Tetramethylheptadecane	**CH**	0.81	
Tetradec-1-ene	**CH**		
2,6,11,15-Tetramethylhexadecane		1.18	
Hexadecan-1-ol	**150.67**	0.59	1.63
Octadec-9-en-1-ol	**683.09**	2.71	**3.51**

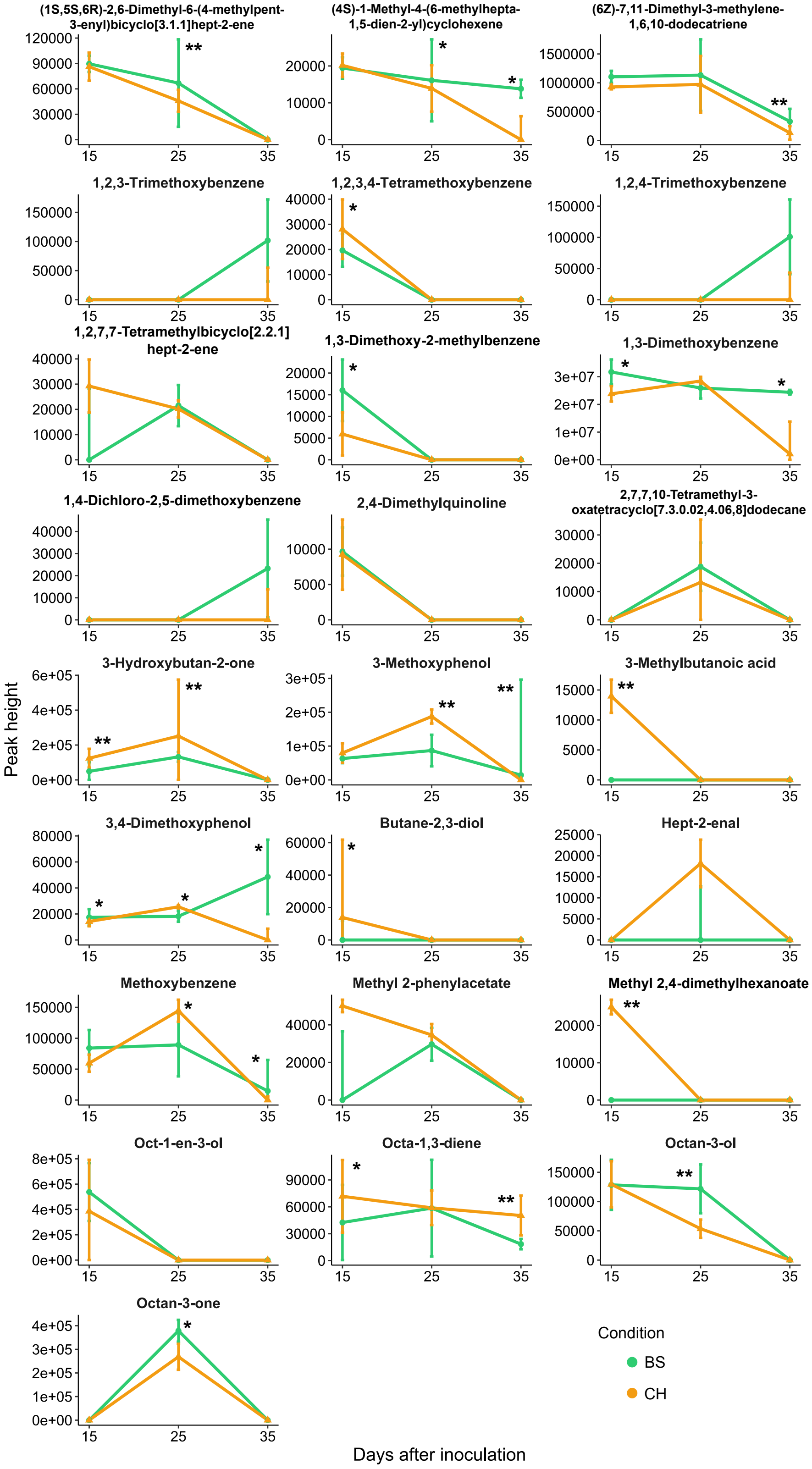

Figure S1: Line plots with the median value and standard deviations of the peak heights of VOCs produced by *P. chlamydosporia* in rice cultures. Abbreviations: VOC = volatile organic compound, CH = Chitosan treatment, BS = Control buffer solution, * = Bayes Factor 3-10 or $\frac{1}{10}$-$\frac{1}{3}$ and ** = Bayes Factor >10 or < $\frac{1}{10}$.

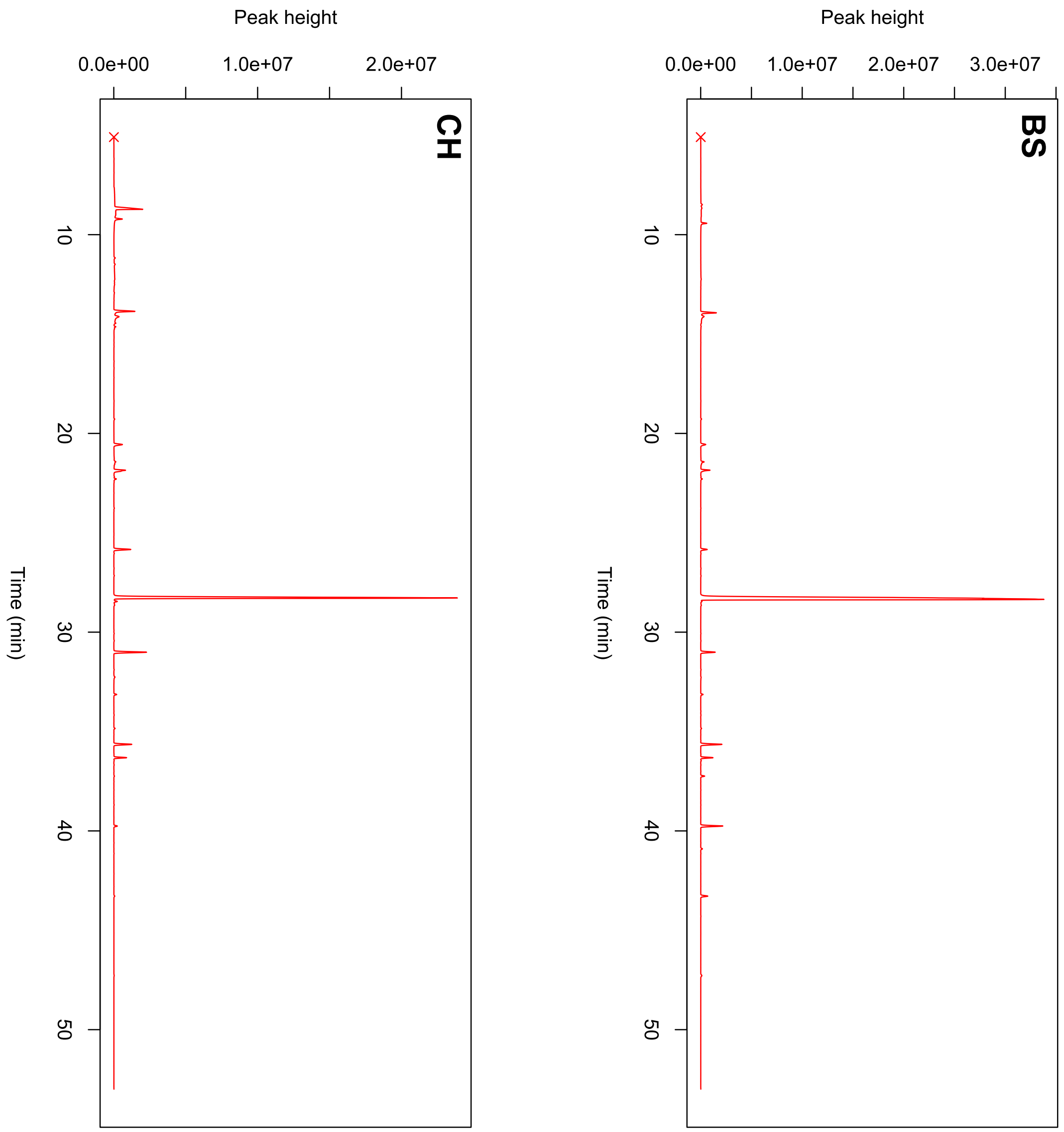

Figure S2: GC-MS chromatogram of headspace volatile compounds produced by *Pochonia chlamydosporia* 15 days after inoculation with control buffer solution (BS) and with chitosan solution (CH).

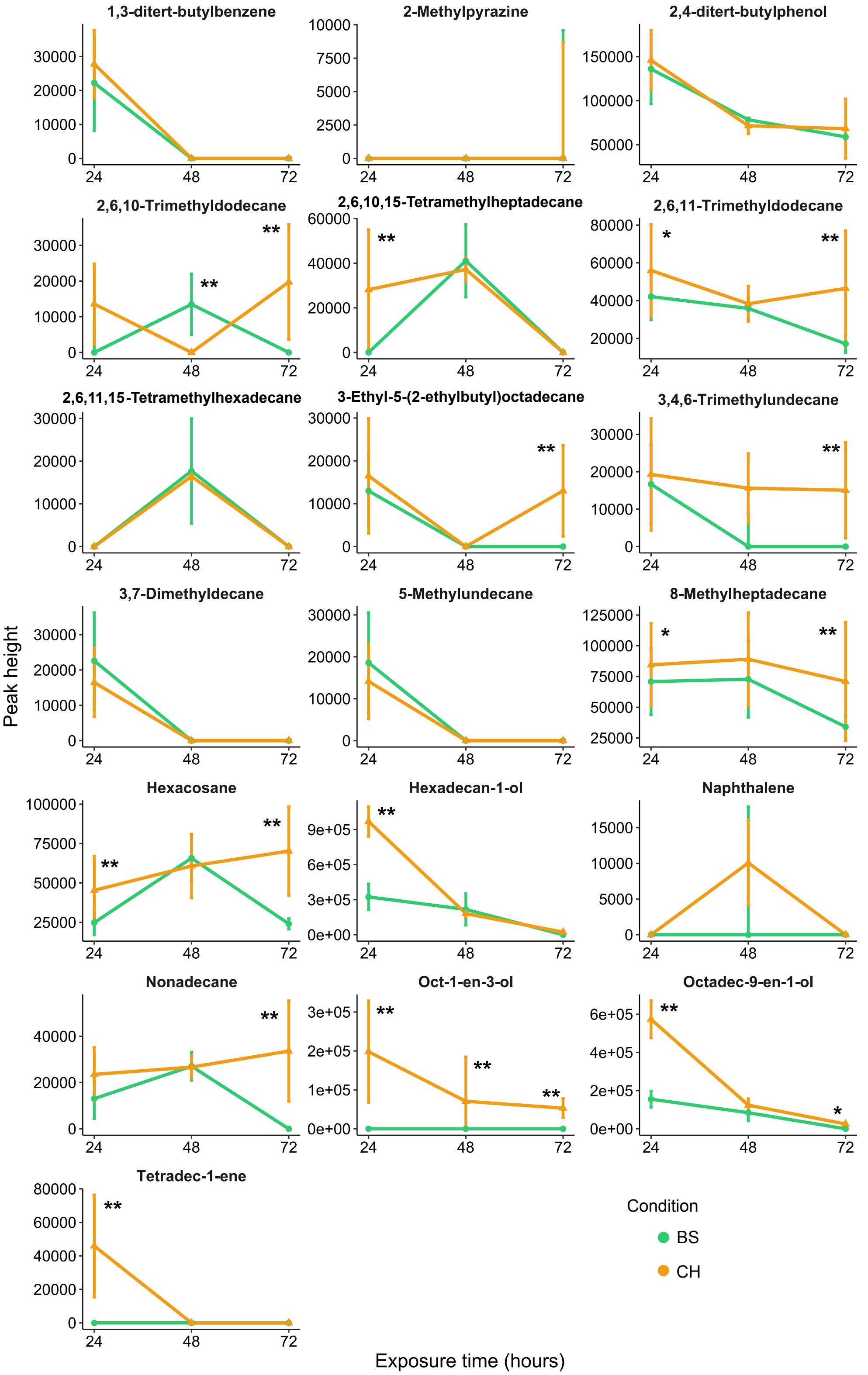

Figure S3: Line plots with the median value and standard deviations of the peak heights of VOCs produced by *P. chlamydosporia* in modified Czapek-Dox broth cultures. Abbreviations: VOC = volatile organic compound, CH = Chitosan treatment, BS = Control buffer solution, * = Bayes Factor 3-10 or $\frac{1}{10}$-$\frac{1}{3}$ and ** = Bayes Factor >10 or < $\frac{1}{10}$.

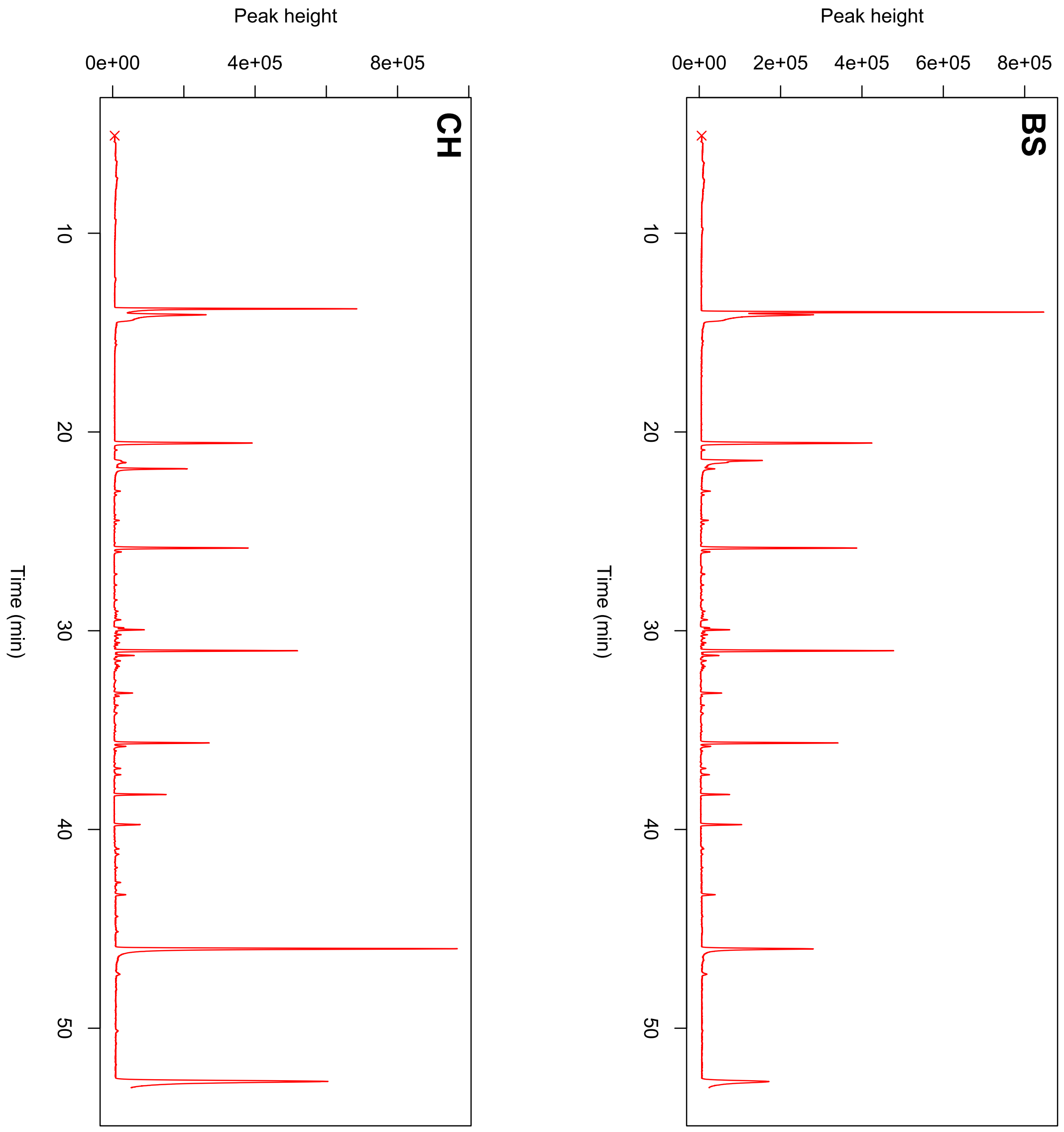

Figure S4: GC-MS chromatogram of headspace volatile compounds produced by *Pochonia chlamydosporia* cultured 5 days in modified Czaped-Dox broth and 24h with control buffer solution (BS) and 24h exposed to chitosan (CH).
